# Neonatal septic arthritis: A tale of two outcomes and literature review

**DOI:** 10.1097/MD.0000000000046328

**Published:** 2025-12-19

**Authors:** Rui Guo, Fei Zhang

**Affiliations:** a Department of Pediatrics, Changde Hospital, Xiangya School of Medicine, Central South University (The First People’s Hospital of Changde), Changde, China.

**Keywords:** diagnosis, management, neonate, omphalitis, septic arthritis

## Abstract

**Rationale:**

Neonatal septic arthritis is a rare disease that can cause severe sequelae if left undiagnosed. It can affect the joints of infants, potentially accelerating osteonecrosis, joint destruction, and permanent deformities. Clinical manifestations are often nonspecific and can vary significantly from one infant to another, contributing to diagnostic challenges and potential delays.

**Patient concerns:**

Two new neonatal patients had fever, red and swollen umbilical cords accompanied by reduced limb movement and swelling.

**Diagnoses and interventions:**

Ultrasound and magnetic resonance imaging showed septic arthritis, blood and umbilical secretion cultures were positive for Staphylococcus. Both the patients underwent arthrotomy, surgical irrigation, and anti-infective treatment.

**Outcomes:**

One patient was diagnosed with septic arthritis of the hip and received treatment only after a 7-day delay from symptom onset, subsequently developing hip joint dislocation as a complication. In contrast, another patient was diagnosed with septic arthritis of the shoulder within 1 day of symptom onset and received prompt treatment, resulting in a favorable prognosis without complications.

**Lessons:**

This case report highlights the significance of early diagnosis and intervention in neonatal septic arthritis. Furthermore, this report underscores the importance of educating parents regarding proper umbilical cord care and vigilant monitoring of newborns to minimize the risk of infections that could progress to septic arthritis. Early medical attention and parental awareness are crucial for reducing morbidity associated with this condition.

## 1. Introduction

Neonatal septic arthritis (SA) manifests as purulent effusion in the joint capsule and synovial inflammation.^[1]^ The synovium is a highly vascularized tissue that lacks a restrictive basement membrane, and is susceptible to bacterial infiltration after pathogenic invasion. The joint cavity environment has low fluid shear stress, which facilitates bacterial adhesion and colonization after invasion. After colonization of synovial fluid, bacteria proliferate rapidly and elicit an acute inflammatory response. If left untreated, elevated cytokine levels produced by the host immune system can significantly damage the infected joints. As infection progresses, joint effusion occurs, which increases intra-articular pressure and inhibits blood and nutrient delivery to the joint, thereby destroying synovium and cartilage.^[2]^ Neonate joints are prone to septic arthritis because they lack a synovial basement membrane and have a rich vascular supply.^[3]^ Septic arthritis in neonates can be caused by different microorganisms such as mycobacteria, viruses, fungi, and bacteria, which promote infection through hematogenous invasion, direct invasion, or spread from adjacent areas.^[4,5]^ In neonates, due to their immature immune systems and the high subcutaneous fat content in their limbs, clinicians may have difficulty detecting localized swelling or warmth at infection sites, leading to delayed diagnosis.^[6]^ Untreated joint and bone infections can cause osteonecrosis, which may result in fractures, limb length discrepancies, deep vein thrombosis, femoral head avascular necrosis, septic shock, sepsis, and in rare cases, death. Therefore, early diagnosis and timely and adequate treatment can improve patient outcomes and prognosis.^[7–10]^ Septic arthritis is typically treated with antibiotics; however, in complex cases, surgical debridement or drainage is performed, whereas it is performed in extremely severe cases.^[11]^ Understanding its epidemiology and clinical features is important for early diagnosis and appropriate treatment strategies to reduce complications and achieve good prognosis. Furthermore, the identification of pathogenic microorganisms is important for confirming the diagnosis and planning antibiotic treatment.^[9]^ Therefore, health care workers in neonatal intensive care units (NICU) should understand the manifestations of bone and joint infections, recognize early symptoms for timely treatment, and prevent long-term sequelae.

This case report describes 2 infants with suppurative arthritis and omphalitis. However, owing to the differences in the timing of diagnosis and therapeutic intervention, 2 dissimilar outcomes have emerged. This demonstrates the significance of early diagnosis and prompt intervention for neonatal suppurative arthritis, specifically with the use of antibiotics.

## 2. Case presentation

Case 1: A 24-day-old female neonate was born in the 39^th^ week of pregnancy *via* cesarean section and weighed 3450 g. The delivery was uncomplicated, with no birth trauma or history of difficult labor. The mother was 24 years old, in her first pregnancy, and had no history of premature membrane rupture or asphyxia. No significant family history of specific diseases or immunodeficiencies was reported.The baby was brought to our hospital with complaints of left leg movement disorder for the last 7 days and swelling on the last 1 day.

Physical examination revealed that the baby was not feeling well and had generalized mottling with a fever of 38.6°C and a respiratory rate of 54 breaths/min, a pulse of 162 beats/min, and a blood pressure of 72/42 mm Hg. Furthermore, redness and swelling of the umbilical stump with foul-smelling yellow discharge were noted in the newborn. Moreover, the movement of the hip joint was limited, and the left lower limb had edema and tenderness. Due to clinical signs suggestive of late-onset sepsis, the neonate was immediately transferred to the NICU. Empirical intravenous antibiotic therapy with piperacillin-tazobactam was initiated to cover common pathogenic bacteria, including Staphylococcus species and Gram-negative bacilli. Laboratory results indicated significantly elevated white blood cell (WBC) count and C-reactive protein (CRP) levels (Table 1 lists the detailed results). Cerebrospinal fluid analysis via lumbar puncture yielded negative findings. Ultrasonography of the left hip joint suggested the presence of a purulent effusion. Eight hours after admission, we promptly adjusted the antibiotic regimen and switched to vancomycin.

**Table 1:** Case presentation.

	Case 1	Case 2
Basic information		
Sex	Female	Male
Gestational age (weeks)	39 + 1	38 + 5
Age (days)	24	12
Infected joint	Left hip	Right shoulder
Laboratory findings		
White-blood-cell count (×10⁹/L)	37.46	13.43
Neutrophil percentage (%)	80.4	49.7
Lymphocyte percentage (%)	12.0	33.1
Platelet count (×10⁹/L)	569	324
C-reactive protein (mg/L)	230.92	80.32
Immature-to-Total neutrophil ratio	0.26	0.66
Serum amyloid A (mg/L)	>350.0	166.69
Joint-fluid culture	Methicillin-resistant *Staphylococcus aureus*	Methicillin-sensitive *Staphylococcus aureus*
Clinical signs and symptoms		
Fever(rectal temperature)	Yes(38.6℃)	Yes(38.7℃)
Feeding refusal	No	No
Neonatal omphalitis	Yes	Yes
Joint swelling	Yes	Yes
Limited range of motion	Yes	Yes
Localized joint tenderness	Yes	Yes
Follow-up outcomes		
Joint deformity	Yes	No
Epiphyseal osteomyelitis	No	No
Joint dysfunction	Yes	No
Skeletal growth arrest	Yes	No

**Figure 1.**
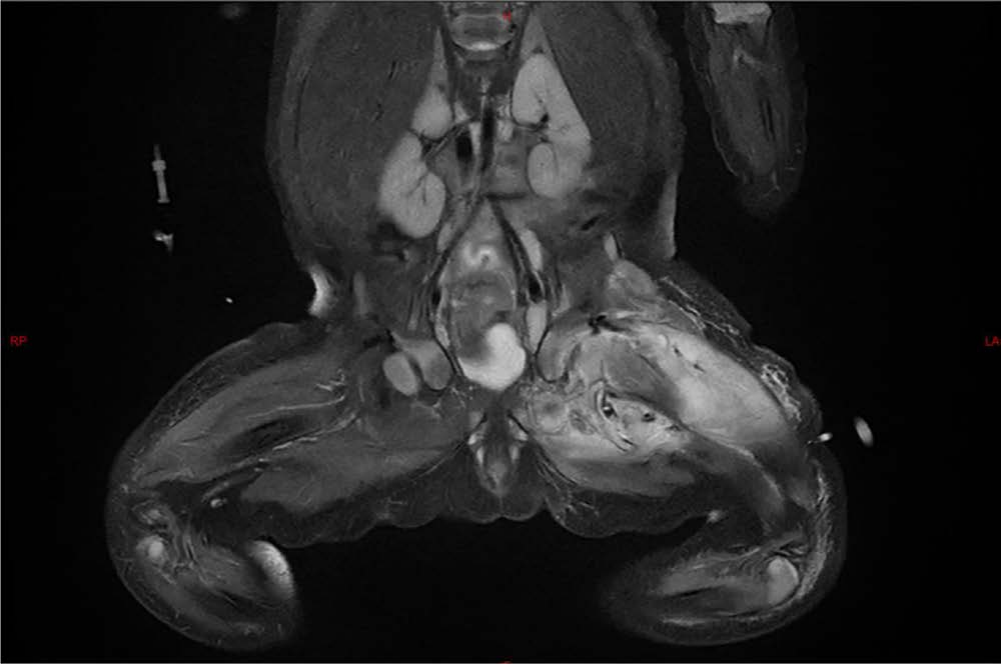
MRI of both hips in a 24-day-old neonate (Case 1). Left thigh abscess formation with infection of the left femoral head and hip joint. MRI = magnetic resonance imaging.

Analysis of blood, joint cavity pus, and umbilical secretion cultures revealed the presence of methicillin-resistant *Staphylococcus aureus* (MRSA). Limb radiography revealed no abnormalities. However, magnetic resonance imaging (MRI) of both hips indicated infectious lesions and abscess formation in both buttocks, the left thigh and a high possibility of infection in the left femoral head and left hip (Fig. 1). Based on the above findings, the final diagnosis was confirmed as septic arthritis of the left hip joint, osteomyelitis of the left femur, and umbilical cellulitis. MRI confirmed the diagnosis by demonstrating an area of altered signal intensity in the left hip joint space and left femoral epiphysis, which resulted in joint space expansion. It also revealed hyperintense signals in the adjacent muscles of the right hemipelvis.

After admission, eft hip joint puncture and catheter drainage were performed, and vancomycin was administered to antimicrobial therapy. On the 7^th^ day of admission, MRI of the hip joint revealed the presence of pus inside and outside the hip joint capsule. Therefore, left hip joint incision and drainage surgery, as well as infection-focused clearance surgery, were performed. The patient remained in the NICU and was treated intravenously with vancomycin for 4 weeks. A reexamination of the routine blood and inflammatory markers showed that the blood culture was negative and the markers were within the normal range. Oral linezolid was administered for 2 weeks. However, at the 3-month follow-up, the baby had a serious sequela of hip dislocation.

Case 2: A 12-day-old male neonate was born at 38^+5^ weeks gestation *via* spontaneous vaginal delivery without history of difficult labor or birth asphyxia. The mother had a preterm membrane rupture and gave birth to a small-for-gestational-age infant weighing 2.3 kg, which falls below the 3rd percentile for infants of the same gestational age and sex. No significant family history of specific diseases or immunodeficiencies was reported. The baby was admitted to the NICU at our facility because of a 4-hour history of fever. No swelling or deformity was observed in any part of the limb. Examination revealed that the baby was very sick, had the following vital signs: a pulse of 180 beats/min, a fever of 38.0 ℃, a respiratory rate of 55 breaths/min, and a blood pressure of 69/39 mm Hg. The infant was generally reactive and showed redness and swelling around the umbilicus, visible granulation tissue at the umbilicus, and yellow discharge from the umbilicus.

After the newborn was admitted, we immediately performed complete blood count (CBC), CRP, procalcitonin (PCT), blood culture, umbilical secretion culture, and cerebrospinal fluid analysis. Within 1 hour, combined antibiotic therapy with cefotaxime and oxacillin was administered for anti-infection treatment. The CBC profile of the baby revealed the following: venous blood WBC count, 13.43 × 10^9^/L; neutrophil percentage, 49.7%; monocyte percentage, 16.8%; hemoglobin concentration: 148.0 g/L, platelet count, 324 × 10^9^/L; Immature-to-Total neutrophil ratio, 0.66; CRP: 80.32 mg/L, serum amyloid A protein: 166.69 mg/L, PCT: 0.40 µg/L. Furthermore, cerebrospinal fluid test results were normal, respiratory virus testing was negative, and chest radiography showed no abnormalities. However, umbilical secretion and blood cultures indicated the presence of methicillin-sensitive *Staphylococcus aureus* (MSSA).

**Figure 2.**
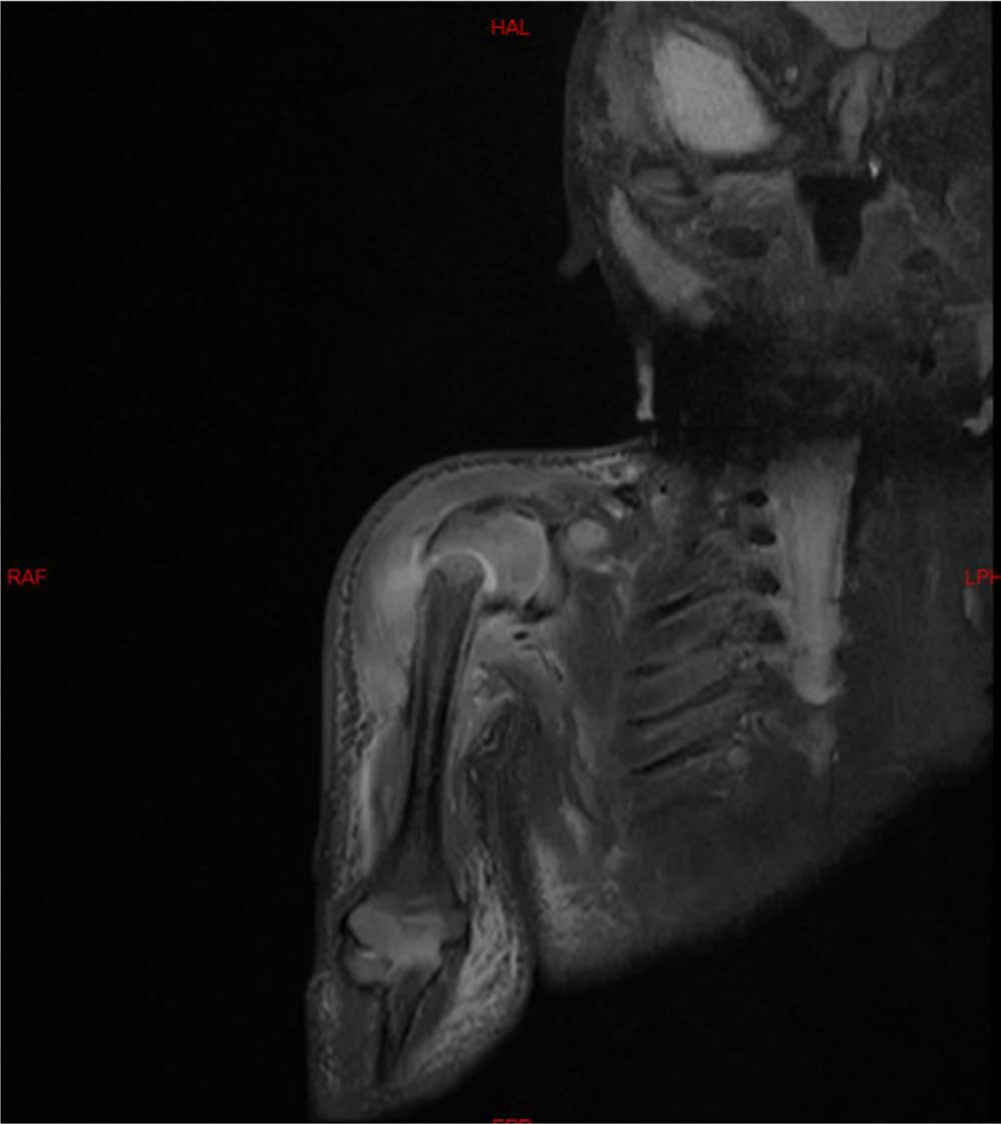
MRI of the right shoulder in a 12-day-old neonate (Case 2). Fracture of the right humeral head epiphysis with associated periarticular soft tissue injury and right shoulder joint effusion. MRI = magnetic resonance imaging.

On the 3^rd^ day of admission, the neonate showed redness and swelling of the right shoulder and less spontaneous right upper limb activity. Emergency shoulder joint ultrasonography revealed separation of the proximal humeral epiphysis and right shoulder joint effusion with surrounding inflammation. Moreover, MRI of the right shoulder joint showed a proximal humeral epiphysis fracture with surrounding soft tissue injury and right shoulder joint effusion (Fig. 2). The following day, ultrasound-guided aspiration of the right shoulder joint cavity was performed, obtaining bloody purulent fluid, which was sent for bacterial culture. This finding confirmed septic shoulder arthritis. After orthopedic consultation, surgical incision, drainage, and irrigation of the joint were subsequently carried out. On the 7^th^ day post-admission, the joint fluid culture yielded MSSA. Cefotaxime was discontinued based on the culture and drug sensitivity results, and oxacillin was continued for 2 weeks, after which the treatment was switched to 3 weeks of oral cefixime treatment. Regular follow-up after discharge revealed no bone or joint abnormalities.

Long-term follow-up was conducted to assess functional outcomes and potential recurrence. At the last follow-up (18 months of age), Case 1 developed a sequelae of left hip dislocation with limb length discrepancy, managed with daytime bracing. The child could walk with support but not independently; however, no recurrent infection was observed, and neurocognitive development was normal. In contrast, Case 2 (11 months of age) exhibited an excellent recovery with full range of motion in the right shoulder and no functional impairments or recurrence.

## 3. Discussion

Bone and joint infections are somewhat uncommon in neonates. The incidence of septic arthritis among children in developed countries is approximately 4 to 10 cases per 100,000 individuals.^[12]^ The 2017 European Society for Paediatric Infectious Diseases Guidelines on Bone and Joint Infections reported an incidence ranging from 1.4 to 22 per 100,000 children in some European countries,^[13]^ while the incidence of SA in neonates is 0.12 to 0.67 cases per 1000 admissions.^[14,15]^ The current literature on neonatal bone and joint infections ranges from case reports to multicenter studies and involves only up to 99 cases.^[16–20]^ Diagnosing of septic arthritis in neonates is more challenging than in older children because of the lack of symptoms and signs. The variation in incidence may be attributed to differences in etiologic diagnostic capacity and surveillance methodologies. However, it is important to note that these data are based on newborns admitted to the hospital and not the overall newborn population. Therefore, the actual incidence may be lower because not all newborns were hospitalized. However, due to the immature immune system of newborns, especially premature infants, the risk of infection is higher; consequently, pyogenic arthritis in newborns, although rare, can be more serious.

The classical presentation of septic arthritis is fever, localizing signs of swelling or pain and limitation of movement or limping. However, these symptoms significantly overlap with those of osteomyelitis and pyomyositis. In neonates, clinical presentations are often atypical, primarily characterized by nonspecific signs such as poor feeding, irritability, lethargy, and/or reluctance to move the affected limb. Consequently, early diagnosis in this population remains particularly challenging. A study of neonatal septic arthritis and osteomyelitis revealed that the most common clinical manifestations include limited activity (64%) and local swelling (60%).^[15]^ Another study of 29 neonates found that fever was the most common clinical symptom (100%), followed by limb movement limitation.^[21]^ Most cases of bone and joint infections occur in primarily healthy children without a predisposing condition. However, during the neonatal period, prematurity, skin infections, bacteremia or candidemia, and a history of central venous catheterization may represent high-risk factors for the development of septic arthritis.^[6,13]^ Case 1 presented with local swelling and movement disorder of the affected limb, whereas case 2 presented with swelling and movement disorder of the affected limb 2 days after fever. Furthermore, both the patients had omphalitis. Cultures from the umbilical secretion, blood, and joint cavity fluid grew the same bacterial species, validating that septic arthritis is caused by bacteria entering the bloodstream *via* the umbilicus and invading the joint cavity. Therefore, it is crucial to provide extra umbilical care during the neonatal period, and physical examinations should be performed.

In neonatal septic arthritis, inflammatory markers, such as Erythrocyte Sedimentation Rate, WBC count, and CRP, are typically significantly elevated.^[21,22]^ In the present study, inflammatory markers showed a variable response. The first infant had a substantial increase in leukocyte, neutrophil, and CRP levels. In contrast, the second infant presented with a normal leukocyte and neutrophil count but a marked elevation in CRP. Therefore, these are not always reliable indicators in neonates because they do not always mount an inflammatory response.^[5]^ It is essential to identify pathogenic bacteria to treat neonatal septic arthritis,^[13]^ and joint effusion aspiration for bacterial culture is the gold standard for diagnosing neonatal septic arthritis. *Staphylococcus aureus* is the most prevalent microorganism involved in bone and joint infections in children at all ages.^[6,13,23]^ A multi-center retrospective study on osteomyelitis and septic arthritis in Chinese neonates and infants revealed that the 3 most common pathogenic bacteria in neonates were MSSA (26.8%), *Escherichia coli* (16.9%), and *Klebsiella pneumoniae* (14.1%).^[20]^ Case 1 had MRSA, while Case 2 had an MSSA infection, which is consistent with previous reports. Generally, a preliminary diagnosis is made based on imaging results before the bacterial culture results arrive. The 2021 U.S. guideline on the diagnosis and management of acute hematogenous osteomyelitis in children recommends that plain radiography should be performed in children with suspected acute hematogenous osteomyelitis, rather than omitting it.^[23]^ Although plain radiography has low sensitivity for detecting osteomyelitis during the initial presentation, this simple, rapid, safe, and relatively inexpensive imaging modality can help exclude other critical differential diagnoses. In both of our cases, plain radiography revealed no significant abnormalities, yet it effectively ruled out fracture-related limb movement impairment and swelling. Ultrasound is a noninvasive and accurate diagnostic tool commonly used in pediatric imaging, especially during the neonatal period.^[24]^ A prospective case-control study quantitatively assessed the alterations in hip joint perfusion in infants with acute septic arthritis using Doppler ultrasonography (Doppler USG).^[25]^ They found that 100% of the hips could obtain Doppler signals and spectra, with significant differences in blood flow parameters between the acute septic arthritis and normal control groups. These hemodynamic parameters can be employed for early detection of hip joint inflammation. Compared with MRI, ultrasound is faster, noninvasive, free of radiation, and does not require sedation or general anesthesia. A study of 33 pediatric patients revealed that before joint incision and debridement for infectious arthritis, advanced imaging studies such as MRI are not required if hip joint effusion has been confirmed *via* ultrasound.^[24]^ Although ultrasonography exhibits high sensitivity in diagnosing septic arthritis, both the European Society for Paediatric Infectious Diseases and the Pediatric Infectious Diseases Society and the Infectious Diseases Society of America consider MRI the most reliable imaging modality for diagnosing bone and joint infections^.[13,23]^

The treatment of neonatal septic arthritis involves multiple disciplines, with antibiotics being the foundation for treatment. Antibiotics should be started early because treatment delays can cause permanent joint and skeletal damage and growth disorders.^[26]^ For the selection of empirical antimicrobial agents for pediatric bone and joint infections (septic arthritis, osteomyelitis), most studies recommend coverage against *Staphylococcus aureus*.^[6,13,23,27]^ This choice should also be based on a comprehensive assessment of local microbiological epidemiology, the child’s age, antibiotic susceptibility patterns, and socioeconomic factors. For children with sepsis and a local community MRSA prevalence of ≥ 10%, the combination of a third-generation cephalosporin (ceftriaxone, cefotaxime, or ceftazidime) with vancomycin is recommended. For children under 3 months of age without sepsis and a local community MRSA prevalence of < 10%, cefotaxime, ceftriaxone, or ceftazidime in combination with an anti-staphylococcal penicillin (such as oxacillin) is suggested.^[28]^ In Case 1, due to the prolonged duration of illness (>1 week), presence of sepsis, and in conjunction with WBC, CRP, and ultrasound findings, the antibiotic regimen was adjusted to vancomycin within 8 hours of admission. In Case 2, since the initial symptom was fever alone, the combination of cefotaxime and oxacillin was selected. Currently, the recommended total treatment duration for hematogenous osteomyelitis caused by Staphylococcus aureus in children is 3-4 weeks on average, while for septic arthritis it is 2-3 weeks.^[13]^ However, prolonged therapy may be required for neonates or immunocompromised children. The French Pediatric Infectious Pathology Group recommends that the shortest duration of antibiotic treatment for infectious arthritis should be 14 days, whereas for neonates, antibiotic treatment should be extended to 4-6 weeks.^[27]^ While general pediatric principles for transitioning to oral therapy exist,^[23,27,28]^ there is no specific consensus on the optimal timing and criteria for this switch in neonates with septic arthritis. The decision is often individualized based on clinical response, causative organism, and physician preference due to the limited high-quality evidence in this vulnerable population.

The main indications for surgical drainage include the presence of large collections, thick pus, joint loculations and pus evacuating into surrounding soft tissues are main indications for surgical drainage. Given the potential for severe sequelae associated with septic arthritis in children, particularly when involving the hip or shoulder joints, joint drainage and irrigation are strongly recommended upon clinical suspicion of SA, especially in neonates and infants under 18 months of age.^[6,13]^ Xu Hongwen et al recommended active surgery for infants in the early disease stage and for those with hip joint infections; however, they suggested conservative treatment for infants whose treatment was delayed for > 15 days.^[29]^ A retrospective study of 189 children under 16 years of age diagnosed with septic arthritis revealed that only 40.7% of the patients underwent arthrotomy or manual irrigation requiring surgical drainage. Compared with culture-negative patients, those with positive cultures required surgical intervention more frequently. Notably, among patients infected with *Staphylococcus aureus*, a higher proportion underwent surgical intervention than conservative management.^[30]^ Another study conducted in Japan identified that the development of sequelae associated with SA was significantly correlated with infection occurring in infants under 1 month of age and delayed surgical intervention (>4 days). The authors recommend that neonates presenting with joint symptoms should undergo surgical drainage as soon as possible, followed by a sufficient course (at least 4–6 weeks) of intravenous antibiotic therapy.^[14]^ In our study, Case 1 was not treated until 1 week after disease onset. The initial treatment included catheter drainage; however, it was ineffective, and arthrotomy with focal debridement was performed 1 week later. Although the neonate received a full course of antibiotic treatment, she still developed a severe complication of hip dislocation during the follow-up. In contrast, Case 2 received early antibiotic treatment and surgical drainage, showed good recovery, and had no sequelae at follow-up, which is consistent with the aforementioned literature. In this regard, we believe that neonates suspected of having septic arthritis should undergo early imaging examinations, particularly MRI. Early surgical incision and drainage may be an effective approach to reduce sequelae of joint damage. For infants who respond poorly to initial needle aspiration, timely follow-up MRI should also be performed to evaluate intra-articular conditions.

For deep-seated joints such as the hip and shoulder, effective drainage can only be achieved through formal arthrotomy, whereas arthroscopy is an alternative method. In neonates, it is difficult to perform traditional arthroscopy, and open surgery has significant risks such as damage to joint structures and postoperative scarring. Recently, a novel needle arthroscopy technique was proposed.^[31]^ It is minimally invasive and not only effectively clears infections but also reduces complications. However, the study sample size was small, and only the shoulder joints were studied.

Furthermore, our study has certain limitations. We did not perform a comprehensive workup for underlying primary immunodeficiencies in these infants. Certain conditions, such as defects in the IL-1R/TLR pathway (e.g., IRAK-4 deficiency), leukocyte adhesion deficiency, or hyper-IgE syndrome (STAT3 deficiency), can predispose individuals to severe and invasive *Staphylococcal* infections. The absence of recurrent infections or a significant family history in our cases makes these deficiencies less likely, but they cannot be entirely ruled out. This highlights an important consideration for clinicians managing neonates with severe bacterial infections and suggests a potential area for future evaluation in cases with a atypical or recurrent course.

## 4. Conclusions

Neonatal septic arthritis is a serious condition that can cause severe complications. The presented cases indicate that septic arthritis should be carefully monitored in neonates with fever, joint swelling, and limb movement disorders, and particularly in those with omphalitis. Early diagnosis, timely and appropriate antibiotic treatment, and surgical intervention are crucial for a good prognosis. Based on our case experience and current research findings, we recommend that surgical drainage be performed as early as possible upon the onset of joint symptoms in neonates diagnosed with septic arthritis, followed by a full course of antibiotic therapy for a minimum of 4 to 6 weeks. The optimal mode and duration of antibiotic therapy, as well as surgical indications, remain controversial and should be individualized according to local protocols and the patient’s clinical profile.

## Acknowledgments

We thank all members of the team involved in patient management and treatment.

## Author contributions

**Conceptualization:** Rui Guo.

**Data curation:** Rui Guo, Fei Zhang.

**Formal analysis:** Rui Guo.

**Investigation:** Rui Guo.

**Project administration:** Rui Guo.

**Visualization:** Rui Guo.

**Writing – original draft:** Rui Guo.

**Writing – review & editing:** Fei Zhang.
